# Photothermally Controlled Drug Release of Poly(d,l-lactide) Nanofibers Loaded with Indocyanine Green and Curcumin for Efficient Antimicrobial Photodynamic Therapy

**DOI:** 10.3390/pharmaceutics15020327

**Published:** 2023-01-18

**Authors:** Bernd Gutberlet, Eduard Preis, Valeri Roschenko, Udo Bakowsky

**Affiliations:** Department of Pharmaceutics and Biopharmaceutics, University of Marburg, Robert-Koch-Str. 4, 35037 Marburg, Germany

**Keywords:** triggered release, antibacterial, aPDT, photothermal therapy, thermosensitive, near-infrared activated

## Abstract

Chronic wound infections with antibiotic-resistant bacteria have become a significant problem for modern healthcare systems since they are often associated with high costs and require profound topical wound management. Successful wound healing is achieved by reducing the bacterial load of the wound and providing an environment that enhances cell growth. In this context, nanofibers show remarkable success because their structure offers a promising drug delivery platform that can mimic the native extracellular matrix and accelerate cell proliferation. In our study, single-needle electrospinning, a versatile and cost-efficient technique, was used to shape polymers into an applicable and homogeneous fleece capable of a photothermally triggered drug release. It was combined with antimicrobial photodynamic therapy, a promising procedure against resistant bacteria. Therefore, poly(d,l-lactide) nanofibers loaded with curcumin and indocyanine green (ICG) were produced for local antimicrobial treatment. The mesh had a homogeneous structure, and the nanofibers showed a smooth surface. Recordings with a thermal camera showed that near-infrared light irradiation of ICG increased the temperature (>44 °C) in the surrounding medium. Release studies confirmed more than 29% enhanced curcumin release triggered by elevated temperature. The antimicrobial activity was tested against the gram-positive strain *Staphylococcus saprophyticus* subsp. *bovis* and the gram-negative strain *Escherichia coli* DH5 alpha. The nanofibers loaded with both photosensitizers and irradiated with both wavelengths reduced the bacterial viability (~4.4 log_10_, 99.996%) significantly more than the nanofibers loaded with only one photosensitizer (<1.7 log_10_, 97.828%) or irradiated with only one wavelength (<2.0 log_10_, 98.952%). In addition, our formulation efficiently eradicated persistent adhered bacteria by >4.3 log_10_ (99.995%), which was also confirmed visually. Finally, the produced nanofibers showed good biocompatibility, proven by the cellular viability of mouse fibroblasts (L929). The data demonstrate that we have developed a new economic nanofiber formulation, which offers a triggered drug release, excellent antimicrobial properties, and good biocompatibility.

## 1. Introduction

Developing efficient strategies to treat infections induced by microbial pathogens is a central focus of modern clinical microbiology. More than anything else, the overuse of antibiotics has led to unstoppable antibiotic resistance that continues to intensify, prolonging hospitalizations, increasing healthcare costs, and finally leading to an increase in deaths. The number of deaths related to global antibiotic resistance will increase to 10 million per year by 2050, which is why there is an urgent need for alternative treatments against resistant bacteria [1]. Favorable conditions for the growth of microorganisms are provided by the loss of skin integrity and exposure of subcutaneous tissue [2]. Wound healing has conventionally been classified into three phases (inflammation, proliferation, and remodeling), and the cellular and molecular biochemical mechanisms involved in wound healing are complex [3]. In particular, chronic wounds are often associated with persistent infections, a prolonged inflammatory phase, and high treatment costs, representing a major burden in this context [4]. Important goals to achieve complete healing of chronic wounds should be a comprehensive wound assessment and treatment of the underlying diseases. Simultaneously, the bacterial load should be suppressed directly at the time of colonization not only to reduce the number of microorganisms but also to prevent biofilm formation [5]. Biofilms also delay wound healing enormously and usually have even higher resistance to antimicrobial agents, hence, accurate treatment with antibiotics or effective alternatives is necessary to prevent the development of antimicrobial resistance (AMR) [6,7,8].

The increase in antibiotic resistance cannot be denied, making innovation, progress and alternative treatment methods against antibiotic-resistant bacteria urgently needed. One mechanism that was discovered before the era of antibiotics is antimicrobial photodynamic therapy (aPDT). This has been neglected for most of the 20th century but has become increasingly important with the emergence of antibiotic resistance [9]. In recent years, more and more research has been conducted on the efficacy of aPDT against microbial infections. In this regard, a variety of photosensitizers have been evaluated on many different microorganisms [10,11,12,13,14,15]. Furthermore, the efficacy of aPDT was also evaluated against coronavirus disease 2019 (COVID-19), in preventing infections and treating infected patients [16,17,18]. Simplified, the function of aPDT depends on three substances: a photosensitizer (PS), light of a specific wavelength (depending on the PS used), and locally available molecular oxygen. By irradiating the PS with the required wavelength, the PS reaches an excited state and can transfer energy or electrons to molecular oxygen. This results in the formation of singlet oxygen in a type II reaction, or reactive oxygen species in a type I reaction, both of which can kill living cells [19,20]. In contrast to antibiotics, which have a specific target, aPDT is a multi-target approach that gives no opportunity for the development of resistances and does not require specific localization of the PS. Therefore, resistance is unlikely to emerge even after multiple applications [20]. Another advantage is the broad spectrum of therapy against gram-positive and gram-negative bacteria, fungi, viruses, and protozoa. However, the combination of aPDT with nanofibers is rather underrepresented in research, although it is gradually attracting more attention [21]. An in vivo study of polyurethane nanofibers with tetraphenylporphyrin reduced wound size and pain in chronic leg ulcers after visible light irradiation [22]. Furthermore, polymer nanofibers loaded with methylene blue were used to study antimicrobial photoactivity against bacteria [23,24] and the healing of infected wounds in a rat model [25].

Indocyanine green (ICG) is a fluorescent, non-toxic heptamethine dye, which has previously been used in aPDT and exhibited good antibacterial and antitumoral properties [26]. Poly(d,l-lactide) (PLA) nanofibers with ICG demonstrated high bacterial reduction after irradiation and a good proangiogenic effect in ovo, emphasizing wound healing promotion [27]. ICG-loaded chitosan/polyvinyl alcohol nanofibers were evaluated against various resistant bacteria, and wound healing was studied in a rat model [28]. Due to its fluorescence in near-infrared light, ICG is commonly used for medical imaging. In this context, ICG-loaded nanofibers were prepared for long-term in situ imaging of cancer cells [29,30].

Curcumin (CUR) is a naturally occurring yellow dye extracted from the rhizomes of turmeric (*Curcuma longa* L.), which has long been used in traditional medicine [31]. Several studies have confirmed the antioxidant, anti-inflammatory, anticancer, and antibacterial effects of CUR but also its low water solubility and poor oral bioavailability [32,33,34,35]. As a result, it has been incorporated into numerous nanoformulations and studied in a broad range of modalities [36,37,38]. Nanofibers have also been loaded with CUR and their antibacterial activity against many different bacteria tested, and the wound-healing effect has been investigated in a rat model [39,40,41].

In this study, we developed PLA nanofibers loaded with ICG and CUR by a single-needle electrospinner, which could be used against infected wounds on the skin, gum, or mucous membranes. Electrospinning is a versatile and cost-effective technique for processing biocompatible polymers such as PLA into a fibrous structure which is applicable in tissue engineering [42] and can be used for local therapy [43]. Due to their structure, nanofibers mimic the physical dimensions of the native extracellular matrix (ECM), which promotes wound healing [44]. By photoactivation of the ICG with infrared light (IR), we can initiate a photothermally triggered release of CUR and thus control the release profile of our fibers. In addition, IR radiation can reach deeper tissue areas with minimal damage to surrounding tissue, which could make it possible to use as an implant and irradiate from outside the body [45]. Subsequently, photoactivation of CUR with blue LED induces the formation of ROS, which can damage the elemental components of bacteria such as lipids and proteins, leading to their death. The combination of photothermal therapy (PTT) with aPDT leads to effective synergistic therapy because the increased temperature enhances the permeability of the bacterial cell wall, which facilitates the uptake of PS and thus may improve the efficiency of aPDT [46,47]. The morphology, drug loading, drug release, and photothermal activity of these dual active nanofibers were analyzed. Antibacterial activity against planktonic gram-positive and gram-negative bacteria and adherent bacteria was evaluated, and capabilities of photothermally triggered drug release were assessed for efficacy. In summary, the characteristics of the proposed novel dual active system indicate excellent potential for application in antimicrobial therapy.

## 2. Materials and Methods

### 2.1. Materials

PLA (molecular weight (M_w_) = 420,000 g/mol, polydispersity = 1.5) was purchased from NatureWorks LLC (Minnetonka, MN, USA). ICG was ordered from Carl Roth GmbH (Karlsruhe, Germany). Curcumin (95%) was purchased from Alfa Aesar (Karlsruhe, Germany). Chloroform was purchased from Acros Organics (Geel, Belgium), and methanol and Tween80 were ordered from VWR (VWR International, Radnor, PA, USA). 3-[4,5-dimethylthiazol-2-yl]-2,5-diphenyltetrazolium bromide (MTT), dimethylsulfoxide (DMSO), Triton X, and Uric acid were purchased from Sigma Aldrich Chemie GmbH (Taufkirchen, Germany). Ultrapure water was used for all the experiments. It was generated by PURELAB^®^ flex 4 equipped with a point-of-use biofilter (ELGA LabWater, High Wycombe, UK).

### 2.2. Bacterial Strains and Media

Prepared glycerol stock cultures of *Staphylococcus saprophyticus* subsp. *bovis* (*S. saprophyticus*, DSM 18669, DSMZ, Braunschweig, Germany) and *Escherichia coli* DH5 alpha (*E. coli*, DSM 6897, DSMZ, Braunschweig, Germany), stored at −80 °C, were thawed one day before the bacterial viability assay. The stocks were cultured in Mueller Hinton broth (MHB, Sigma Aldrich Chemie) on an orbital shaker (Compact Shaker KS 15 A, equipped with Incubator Hood TH 15, Edmund Bühler, Bodelshausen, Germany) set at 200 rpm and 37 °C.

### 2.3. Cell Lines and Media

Mouse fibroblast cell line (L929) was purchased from DSMZ (Braunschweig, Germany). The cells were cultivated in RPMI 1640 cell culture medium from Capricorn Scientific (Ebsdorfergrund, Germany) at 37 °C and 5% CO_2_ under humid conditions. The media were supplemented with 10% fetal calf serum (PAA, Cölbe, Germany) and 1% antibiotic/antimycotic solution (100×, Capricorn Scientific GmbH, Germany).

### 2.4. Light Source

The irradiation experiments presented in this work were performed with a Weberneedle Endo Laser (Weber medical GmbH, Lauenförde, Germany) and a prototype low-power LED device consisting of an array of light-emitting diodes designed to fit multiwell plates (Lumundus GmbH, Eisenach, Germany). The Weberneedle was equipped with a laser module (810 nm, 500 mW) and an optical fiber. The LED device was set at 100 mA at a wavelength of 457 nm. By adjusting the distance between the irradiated surface and the optical fiber, the diameter of the light spot became suitable for the irradiation of single samples. Radiant exposure was calculated based on the irradiance and irradiation time.

### 2.5. Preparation of Nanofibers

The nanofibers were prepared by single-needle electrospinning using a custom-made electrospinner. Briefly, 6 wt% PLA (relative to the mass of total spinning solution) alone, or with either 3 wt% ICG (relative to PLA mass) or 10 wt% CUR (relative to PLA mass), or with both were dissolved in chloroform:methanol (2:1) by continuous stirring on a magnetic stirrer at room temperature (RT) for 3 h. Thus, four different samples were obtained (Table 1). The solution was aspirated with a syringe, equipped with a 21G needle, and clamped in a syringe pump. The spinning process ran for 1 h at RT with a vertical needle movement parallel to the drum collector to produce a homogeneous fiber mat. The following process parameters were used for electrospinning: voltage 15 kV, distance between collector and needle tip 10 cm, drum collector 200 rpm, and flow rate of the syringe pump 1 mL/h.

### 2.6. Scanning Electron Microscopy (SEM) of the Fibers

The morphology of the nanofibers was investigated by SEM (Hitachi S-510, Hitachi-High Technologies Europe GmbH, Krefeld, Germany). Nanofiber samples were punched out and were provided with conductive double-sided adhesive carbon tabs to fix onto aluminum pin stubs. The samples were sputter-coated with a gold layer (three times 10 mA for 1 min) using Edwards S150 Sputter Coater (Edwards Vacuum, Crawley, UK) and were scanned using an acceleration voltage of 5 kV and a secondary electron detector. By analyzing three independent SEM images of each sample, the mean fiber diameter was determined. For this purpose, each SEM micrograph was evaluated with “DiameterJ”, a plugin (version 1.018, Nathan Hotaling) created for ImageJ (version 1.53a, National Institutes of Health, Bethesda, MD, USA). The fiber diameter distribution was calculated as a frequency of the fiber diameter and is expressed in percentage.

### 2.7. Drug Loading

Nanofiber samples of each formulation (PLA.NF.ICG, PLA.NF.CUR, PLA.NF.ICG.CUR) were punched out with a diameter of 16 mm and dissolved in chloroform:methanol (2:1, *v*/*v*) assisted by vortexing. The concentration of loaded ICG and CUR was determined by measuring the absorbance with a microplate spectrophotometer (Multiskan GO, Thermo Scientific, Waltham, MA, USA) at λ = 424 nm (CUR) and λ = 797 nm (ICG). By using a calibration curve, the content of CUR and ICG was calculated and related to the area of the nanofiber mesh by applying the following Equation (1):

(1) $$p_{CUR}\;\left[\frac{mg}{cm^{2}}\right]=\frac{m_{Cur}\left[mg\right]}{A_{mesh}\left[cm^{2}\right]}\;or\;\;p_{ICG}\;\left[\frac{mg}{cm^{2}}\right]=\frac{m_{ICG}\left[mg\right]}{A_{mesh}\left[cm^{2}\right]}$$

where $p_{CUR}\;or\;p_{ICG}$ is the content of CUR or ICG, respectively, $m_{CUR}\;or\;m_{ICG}\;$ is the mass of incorporated CUR or ICG, respectively, and $A_{mesh}$ is the area of the nanofibrous mesh.

### 2.8. Photothermal Activity

Nanofiber samples of PLA.NF, PLA.NF.ICG, PLA.NF.CUR, and PLA.NF.ICG.CUR with a diameter of 16 mm were punched out and fixed onto the bottom of 24-well cell culture plates (TC plate, Standard, F, Sarstedt AG & Co. KG, Nümbrecht, Germany). After a 30 min incubation time in 250 µL phosphate-buffered saline (PBS, pH 7.4) at RT, the samples were irradiated with the previously described laser module and the setup of the optical fiber (Section 2.4). The temperature change related to irradiation was analyzed at several time intervals by thermal imaging (FLIR ONE Pro, FLIR Systems, Inc., Wilson-ville, OR, USA).

### 2.9. In Vitro Drug Release Studies

As previously described (Section 2.7), the nanofiber mesh was precisely cut into round pieces with a diameter of 16 mm. All samples were separately fixed onto the bottom of a 6-well cell culture plate (TC plate, Standard, F, Sarstedt AG & Co. KG, Nümbrecht, Germany), and 1.5 mL of 1% Tween80 in PBS (pH 7.4) was pipetted into each well to provide sink conditions [48]. After 30 min of incubation under light exclusion and at RT, a sample was taken from the solvent, and the fibers were irradiated with the laser module (Section 2.4) for 30 min. After irradiation, another sample was taken, and the fibers were incubated for another 30 min before a final sample was collected. Regarding the sampling, the well plates were shaken, and 150 µL was taken and replaced with fresh solvent. Samples were pipetted into a 96-well plate (Microtest Plate, F, Sarstedt AG & Co. KG, Nümbrecht, Germany), and the absorption was determined with a microplate spectrophotometer (Multiskan GO, Thermo Scientific, Waltham, MA, USA) at λ = 424 nm. The CUR concentration was calculated using a calibration curve.

### 2.10. Antimicrobial Photodynamic Activity

#### 2.10.1. Planktonic Bacteria

The nanofibers were incubated with bacterial suspensions and irradiated to determine the antimicrobial activity. The growth of both bacterial suspensions (*S. saprophyticus* and *E. coli*; Section 2.2) was stopped by placing them on ice at an optical density (OD_600_ at λ = 600 nm) of 0.4 measured with a spectrophotometer (Shimadzu UV mini-1240, Kyoto, Japan). The nanofiber samples with a diameter of 16 mm were placed on the bottom of a 24-well cell culture plate (TC plate, Standard, F, Sarstedt AG & Co. KG, Nümbrecht, Germany) and fixed to the bottom by a Teflon ring. 250 µL of the bacterial suspensions were pipetted into each well, and the fibers were incubated under light protection for 30 min at RT. Each sample was irradiated with an IR laser (λ = 810 nm, 500 mW) and either with or without blue LED (λ = 457 nm, 100 mA) for 30 min, respectively. Afterwards, the treated suspensions were diluted several times with MHB and plated onto Mueller Hinton II agar plates (BD, Heidelberg, Germany). The agar plates were incubated for 18 h at 37 °C and 90% relative humidity. After this time, the viable colonies were counted and the colony-forming units were calculated per milliliter (CFU/mL). The conditions were the same for both bacterial strains. PLA.NF.ICG, PLA.NF.CUR, PLA.NF.ICG.CUR were compared to unloaded PLA.NF and dark control groups without irradiation. The experiments were performed in triplicates under light-protected conditions.

#### 2.10.2. Adhered Bacteria

A bacterial suspension of *S. saprophyticus* was prepared as described above (Section 2.2) and was diluted with fresh medium to an OD_600_ of 0.1. Titanium discs (Ti-discs; 1 cm^2^) were placed in a 24-well cell culture plate (TC plate, Standard, F, Sarstedt AG & Co. KG, Nümbrecht, Germany) and covered with 2 mL of this suspension. Afterward, the well plate was incubated for 48 h at 37 °C and 90% RH. The Ti-discs were immersed thrice in PBS (pH 7.4) and placed in a new well to remove non-adherent bacterial cells. Nanofiber samples were placed on the adhered bacteria formed on the Ti-discs, and 250 µL of PBS was added. After 30 min incubation under light protection and at RT, each sample was irradiated with an IR laser (λ = 810 nm, 500 mW) and either with or without blue LED (λ = 457 nm, 100 mA) for 30 min, respectively. The nanofibers were removed, 1 mL trypsin-EDTA (0.5%, Capricorn Scientific GmbH) was added, and the samples were gently shaken (100 rpm) at RT for 20 min using an orbital shaker (Compact Shaker KS 15 A, Edmund Bühler GmbH) to detach the bacteria from the surface of the Ti-discs. The solutions were serially diluted with MHB and plated onto Mueller Hinton II Agar plates (BD, Heidelberg, Germany). After incubating the plates for 18 h at 37 °C and 90% relative humidity, the viable colonies were counted, and the colony-forming units per square centimeter were calculated (CFU/cm^2^).

#### 2.10.3. Qualitative Assessment of Surface Eradication of the Bacteria via SEM

Changes in the adhered bacteria after treatment with the nanofibers were visualized by SEM. For this purpose, the titanium discs on which the bacteria were grown were washed thrice with PBS to remove non-adhered bacteria. The samples were then incubated in 2.5% glutaraldehyde-PBS solution for 2 h at room temperature (RT). After that, the bacteria were dehydrated in increasing ethanol concentrations of 30, 50, 70, 90, and 100% for 20 min each on ice. Then, the samples were dried in a solution of ethanol absolute and hexamethyldisilazane (1:1) for 20 min on ice. Subsequently, the Ti-discs were incubated in pure hexamethyldisilazane solution for 20 min on ice. Following the sample preparation, they were fixed onto aluminum pin stubs using conductive double-sided adhesive carbon tabs and sputter-coated thrice with a gold layer (10 mA for 1 min). The titanium discs were recorded using an acceleration voltage of 5 kV and a secondary electron detector.

### 2.11. Biocompatibility Studies

Nanofiber samples (PLA.NF, PLA.NF.ICG, PLA.NF.CUR, and PLA.NF.ICG.CUR) with a diameter of 10 mm were cut out and placed in a 48-well plate (TC Plate, Standard F, Sarstedt). The samples were fixed to the bottom with a silicone ring. Cells were seeded at a concentration of 25,000 cells/well and a volume of 0.5 mL/well. The well plate was incubated overnight at 37 °C and 5% CO_2_ so that the cells could adhere to the fibers. After 20 h, the medium was removed, and 250 µL MTT reagent (concentration = 0.2 mg/mL) was added to each well. The samples were incubated at 37 °C and 5% CO_2_ for 4 h. Afterwards, the MTT reagent was removed carefully, and the formazan crystals were solved in 0.5 mL DMSO by placing the well plate in an incubator shaker (IKA KS 4000 ic control, IKA-Werke GmbH & Co. KG, Staufen, Germany) at 150 rpm and 37 °C for 20 min. 200 µL of this solution was pipetted in a 96-well plate (NUNC, Thermo Fischer Scientific GmbH, Germany), and the absorbance was measured at 570 nm using a plate reader (Fluostar OPTIMA BMG Labtech, Germany). The values of cells incubated with medium represent 100% viability, and 0.1% Triton X was used as a positive control. As the nanofiber samples are deposited on aluminum foil, the foil was also investigated without fibers (Blank).

### 2.12. Statistical Analysis

All experiments were performed in triplicates, and the results are presented as mean ± standard deviation (SD) unless explicitly stated otherwise. Two-tailed t-test and two-way ANOVA test were performed to determine significance, and probability values of *p* < 0.05 were considered significant. Statistical differences are denoted as “*” *p* < 0.05, “**” *p* < 0.01, “***” *p* < 0.001, and “****” *p* < 0.0001.

## 3. Results and Discussion

### 3.1. Morphology of the Nanofibers

The nanofibers were produced via electrospinning, a commonly used and versatile technique for producing nanoscale fibers, using a custom-made single-needle electrospinner [46]. Many parameters significantly influence the process, such as electric field strength, polymer concentration, the flow rate of the polymer solution, and distance of the needle to the collector. Nevertheless, various polymers can be successfully spun to form fibrous meshes with different thicknesses [49]. In this study, PLA nanofibers with varying drug combinations were prepared (Table 1). The PLA concentrations were 6% based on the mass of the total spinning solution, and the drug concentrations were based on the PLA mass.

**Figure 1:**
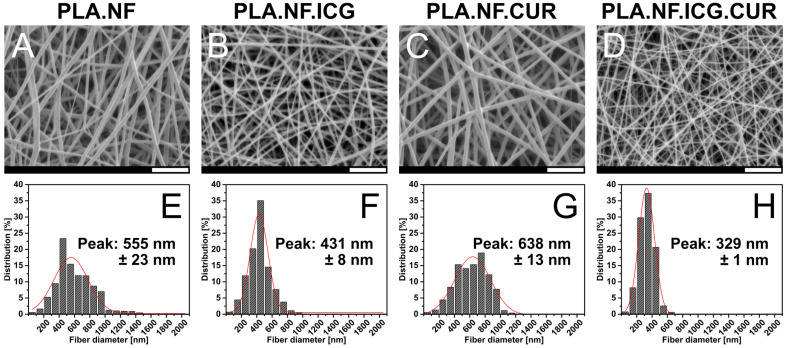
SEM micrographs of the prepared formulations (**A**–**D**) with their fiber diameter size distribution (**E**–**H**). PLA.NF (**A**,**E**), PLA.NF.ICG (**B**,**F**), PLA.NF.CUR (**C**,**G**), and PLA.NF.ICG.CUR (**D**,**H**). Scale bars represent 7 µm.

The morphology of the prepared nanofibrous meshes was examined by SEM, and the micrographs are presented in Figure 1A–D. All formulations showed a fibrous structure with a smooth surface. No beads or crystals could be seen, indicating that most of the drugs were inside the PLA matrix. All formulations showed a homogeneous size distribution with low SD presented in Figure 1E–H. A pronounced decrease in the fiber diameter loaded with both drugs (PLA.NF.ICG.CUR, 329 nm) was observed compared to the unloaded ones (PLA.NF, 555 nm), which could be attributed to the higher conductivity of the spinning solution [50]. The diameters of the single loaded fibers differed only slightly from that of the unloaded fibers and can be explained by measurement inaccuracies and minor changes in conductivity (PLA.NF.ICG, 431 nm; PLA.NF.CUR, 638 nm). All fibrous meshes looked complete and homogeneous. The samples are in the nanometer range and can be used for further investigations.

### 3.2. Photothermal Activity

**Figure 2:**
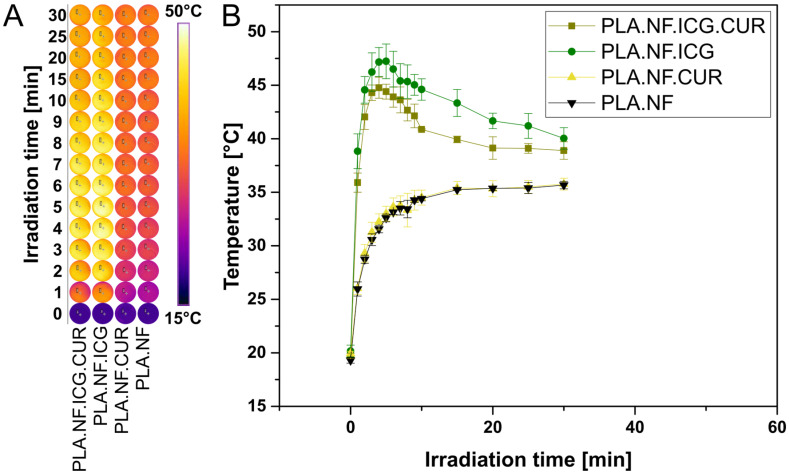
(**A**) Thermal images of each formulation during 30 min irradiation (810 nm) and (**B**) graphical representation of the results.

Thermal imaging was used to evaluate the temperature changes during irradiation and the results are presented in Figure 2. ICG has a wide range of applications [51]. It not only has excellent properties for use in photothermal therapy (PTT) but can also be used for photodynamic therapy (PDT). Both PDT and PTT proved to be effective strategies to eradicate bacteria without risking AMR [52]. Thus, ICG is not only beneficial for photothermally triggered release but also could increase bacterial reduction by combining PTT and PDT. During the irradiation of PLA.NF.ICG.CUR and PLA.NF.ICG, the temperature increased rapidly and reached the maximum after only 4 and 5 min, respectively (44.8 and 47.2 °C). This represents an increase in temperature of 25.1 and 27.0 °C, respectively. Afterwards, the temperature dropped again in both cases but could be maintained above 38.8 °C during the entire irradiation period of 30 min. The slightly higher temperature rise of PLA.NF.ICG during irradiation compared to PLA.NF.ICG.CUR, although they have an almost identical ICG concentration, might indicate photobleaching due to the additional CUR. PLA.NF.CUR and PLA.NF heat up slightly due to the high energy of the infrared light of the laser. However, no difference is seen between the two formulations, indicating that the intense photothermal effect comes exclusively from the ICG.

Several studies reported the effective use of PTT to significantly reduce bacterial viability, especially in combination with other agents, as the photothermal effect is expected to increase bacterial uptake by enhancing the permeability of the bacterial cell wall [46]. However, the operating temperatures were in the range of 55 to 60 °C, potentially causing damage to the treated area and surrounding skin [53,54]. Although the temperature could possibly rise higher without the required bacterial medium, we stay well below 60 °C, so our formulation could provide a valid alternative.

### 3.3. Drug Loading and In Vitro Drug Release

First, the overall concentration of ICG and CUR was determined photometrically and related to the area of the fibrous mesh. The results are presented in Table 2. The ICG concentration of the double-loaded fibers (PLA.NF.ICG.CUR) is almost identical to that of the fibers loaded only with ICG (PLA.NF.ICG). In addition, the SD is very low, which indicates good reproducibility of the production and loading. The CUR concentration of PLA.NF.ICG.CUR is significantly lower than that of the fibers loaded only with CUR (PLA.NF.CUR), which can be attributed to the narrower fiber mat of PLA.NF.CUR. Because of the higher conductivity of the spinning solution of PLA.NF.ICG.CUR, there is a greater electric current during electrospinning which results in large accumulations of charge on the fibers, increasing the electrical force and the fibers’ deposition area [55]. In contrast, the fiber mat of PLA.NF.ICG had a similar size to that of PLA.NF.ICG.CUR, indicating a similar conductivity of the spinning solution which was confirmed by the smaller fiber diameter and the almost identical concentration per area. Nevertheless, the SD of the CUR concentration is also very low, indicating a reproducible process. Moreover, the curcumin distribution in the nanofibers was homogeneous (see Figure S2).

**Figure 3:**
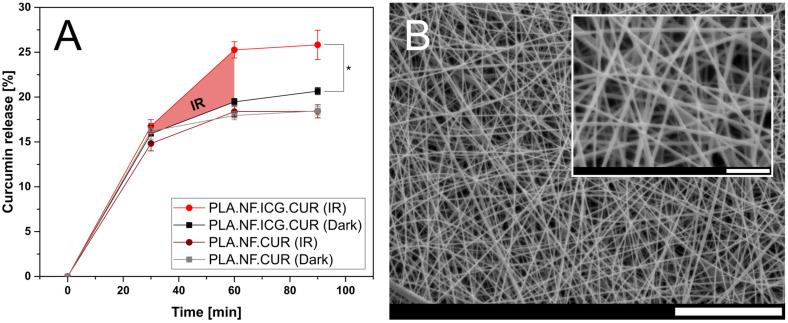
Curcumin release of PLA.NF.ICG.CUR and PLA.NF.CUR plotted as a function of time (**A**), Dark: 90 min incubation under light exclusion, IR: 30 min incubation, followed by 30 min irradiation (810 nm), and 30 min incubation. SEM micrographs of PLA.NF.ICG.CUR after irradiation (**B**). Scale bars represent 20 µm and 4 µm for the inset. Statistical differences are denoted as “*” *p* < 0.05.

The photothermally triggered release of CUR from PLA.NF.ICG.CUR and PLA.NF.CUR was photometrically analyzed under sink conditions. Additionally, the fibrous mesh was examined for morphological changes after irradiation with SEM (Figure 3). A burst release of CUR was observed for the first 30 min of incubation because of the high surface area of the fibers and parts of CUR deposited on the surface [39,56,57]. After irradiation of PLA.NF.ICG.CUR, significantly more CUR was released compared to the non-irradiated fibers. Remarkably, when the irradiation was interrupted, the CUR release almost stopped, indicating good incorporation of the drugs into the fibers and possibly enabling long-term treatment. The total amount of released CUR significantly increased by more than 29% with a 30 min irradiation step (810 nm). The morphological structure of the fibrous mesh was preserved even after irradiation, which could still promote wound healing after the reduction of bacterial load. No significant difference was observed between the irradiated and non-irradiated samples for the fibers loaded with CUR only (PLA.NF.CUR), proving that the increased release is purely attributed to the activated ICG and that the laser alone does not influence the release. The higher drug release of the non-irradiated double-loaded fibers compared to those loaded only with CUR could be explained by the smaller fiber diameter and larger surface area of the PLA.NF.ICG.CUR. The released curcumin can form ROS under irradiation with the blue LED visible in both PLA.NF.ICG.CUR and PLA.NF.CUR (see Figure S1). PLA.NF.ICG.CUR produces more ROS than PLA.NF.CUR, while PLA.NF.ICG and PLA.NF show no changes during blue LED irradiation. The UA absorption of PLA.NF.ICG is lower than PLA.NF because the previous IR irradiation leads to a photoreaction of ICG and a small amount of ROS.

Triggered drug release by near-infrared light was first evaluated on other drug delivery systems [58]. Xi et al. also studied curcumin release from nanofibers using IR irradiation, but were only able to increase the release by 28% after four irradiation cycles within 240 min [59]. Furthermore, there was a considerable continuous release between irradiations. Zhao et al. displayed the influence of pH and temperature on the release of doxorubicin in nanofibers, and they were able to increase the release rate by IR irradiation [60]. The significantly enhanced drug release of PLA.NF.ICG.CUR (29%) and its controlled release profile might avoid any additional physical intervention and offer novel and future-oriented possibilities.

### 3.4. Antimicrobial Photodynamic Activity

The principle of photodynamic therapy was discovered many years ago and has been known for a long time. However, proper dosimetry is still a challenge to this day. The success of antimicrobial photodynamic therapy (aPDT) depends on multiple factors. Many different parameters have been studied, and their influence evaluated, however, success depends mainly on the irradiance and radiant exposure or the overall dosimetry [61,62]. There are some variations depending on the photosensitizer used, although the multifactorial concept of dosimetry should be considered in any experiment [63].

The cell toxicity of the photo-activated curcumin against bacteria is based on several different modes of action, such as DNA damage, destruction of the cytoplasmic membrane, or inactivation of transport proteins [64]. CUR shows excellent photodynamic activity but, as mentioned above, is not suitable for therapy because of its low water solubility and bioavailability. These problems can be counteracted, and CUR can be incorporated into nanoscale drug delivery systems [36,37,65].

#### 3.4.1. Planktonic Bacteria

**Figure 4:**
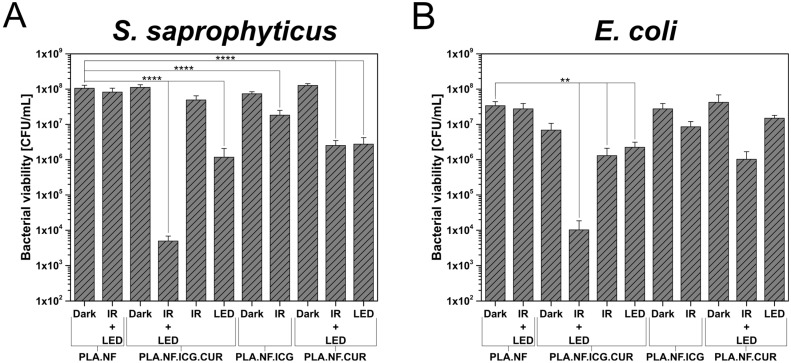
Bacterial viability of *Staphylococcus saprophyticus* subsp. *bovis* (*S. saprophyticus*; (**A**)) and *Escherichia coli* DH5 alpha (*E. coli*; (**B**)) on the different formulations of the produced nanofibers after irradiation with a laser module (IR: 810 nm) and an LED panel (LED: 457 nm) for 30 min each. Unloaded fibers (PLA.NF) were used as the negative control and dark represents unirradiated bacteria. Probability values of *p* < 0.05 were considered significant. Statistical differences are denoted as “**” *p* < 0.01 and “****” *p* < 0.0001.

In this study, the bacteria were placed on the different formulations of the prepared nanofibers and either kept in the dark or irradiated. The results against gram-positive bacteria are presented in Figure 4A and those against gram-negative bacteria in Figure 4B. In both cases, no significant dark toxicity was observed for any of the formulations. In addition, the influence of the light source is negligible, as no changes in the viability of either germ could be detected when comparing dark and irradiated unloaded fibers. In contrast, the PLA.NF.ICG.CUR irradiated with both wavelengths reduced the gram-positive bacterial viability significantly by ~4.4 log_10_ (99.996%) and the gram-negative bacterial viability significantly by >2.8 log_10_ (99.852%). Irradiation of this formulation with the IR laser alone showed only a marginal bacterial reduction, which implies that the concentration of ICG is not enough to kill the bacteria via the photothermal or photodynamic reaction. Irradiation of PLA.NF.ICG.CUR with the blue LED alone significantly reduced gram-positive bacteria by <2.0 log_10_ (98.952%) and gram-negative bacteria by <0.5 log_10_ (67.415%). As described in Section 3.3, there is a burst release of CUR at the beginning, which could explain this bacterial reduction. The results demonstrate that we were able to more than double the bacterial reduction by irradiating with both wavelengths. This might be due to the increased amount of released CUR, but also because of the photothermal effect, which could enhance the uptake of CUR into the bacterial cells. Irradiation of the other formulations gave the expected results. PLA.NF.ICG showed similar results as the single IR irradiation of PLA.NF.ICG.CUR, and PLA.NF.CUR gave results related to the irradiation of PLA.NF.ICG.CUR with the LED alone. It was observed that CUR showed slightly lower antibacterial activity against gram-negative bacteria. Furthermore, the previous IR irradiation of PLA.NF.CUR exhibited no enhancement of the antibacterial effect in gram-positive *S. saprophyticus*, whereas this was the case in gram-negative *E. coli*. As shown in Section 3.2, IR irradiation alone leads to a slight increase in the temperature of the surrounding medium even without ICG. This could lead to a mild increase in the release of the curcumin, which could explain the higher bacterial reduction of PLA.NF.CUR after previous IR irradiation. Only the combination of both photosensitizers and both wavelengths showed a bacterial reduction of 99.996%, qualifying the formulation as antibacterial, according to the American Society for Microbiology.

#### 3.4.2. Adhered Bacteria

**Figure 5:**
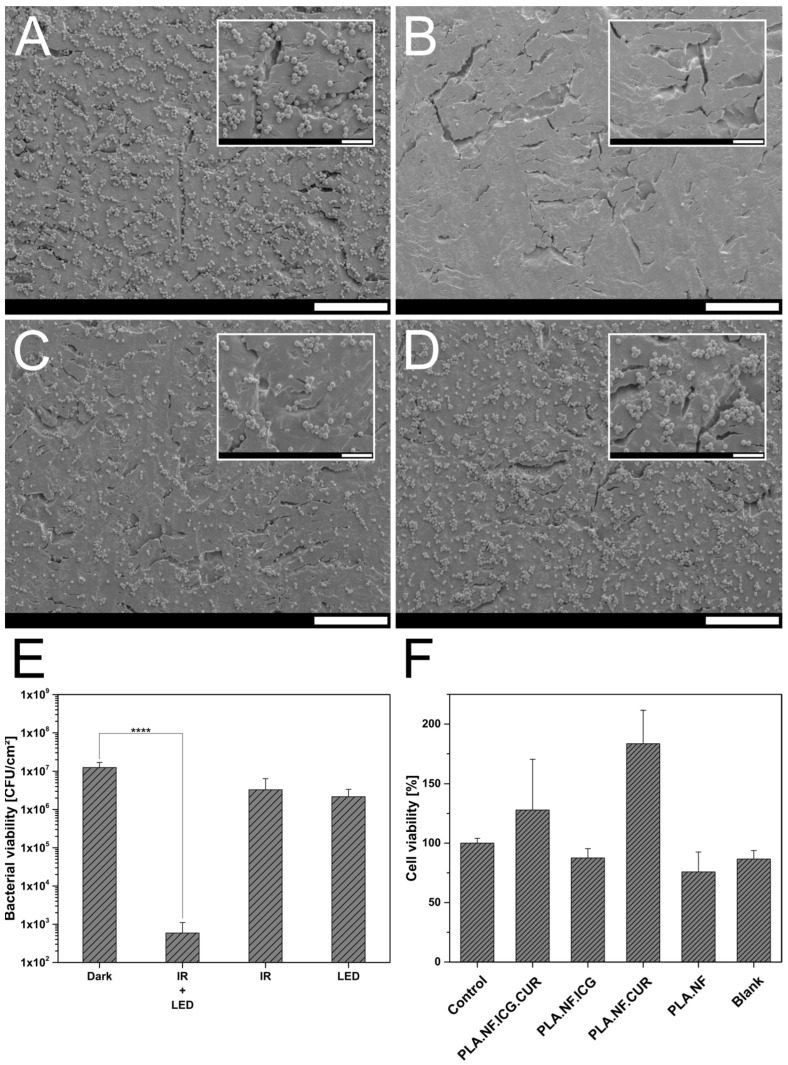
(**A**–**D**): SEM images of the different irradiated adhered bacteria from *Staphylococcus saprophyticus* subsp. *bovis* (*S. saprophyticus*) treated with PLA.NF.ICG.CUR. (**A**) unirradiated bacteria, (**B**) irradiated with a laser module (IR: 810 nm) and an LED panel (LED: 457 nm) for 30 min each, (**C**) irradiated with a laser module (IR: 810 nm) for 30 min, (**D**) irradiated with an LED panel (LED: 457 nm) for 30 min. Scale bars represent 30 and 7 µm for the insets. (**E**) Bacterial viability of treated adhered bacteria from *Staphylococcus saprophyticus* subsp. *bovis* (*S. saprophyticus*) treated with PLA.NF.ICG.CUR after irradiation with a laser module (IR: 810 nm) and an LED panel (LED: 457 nm) for 30 min each. Dark represents unirradiated bacteria. Probability values of “****” *p* < 0.0001 were considered significant. (**F**) Cellular viability of L929 cells after incubation with PLA.NF.ICG.CUR, PLA.NF.ICG, PLA.NF.CUR, and PLA.NF. An empty well was used as control and empty aluminum foil on which the fiber samples were supported was used as blank.

As the treatment of adhered bacteria and a forming biofilm is always challenging because of beneficial conditions for the bacteria, the produced nanofibers were placed on top of an adhered bacterial film and either kept in the dark or irradiated. After treatment, the bacteria were detached and their survivability determined, and the results are presented in Figure 5E. PLA.NF.ICG.CUR irradiated with both wavelengths reduced the bacterial viability of the bacteria significantly by >4.3 log_10_ (99.995%). The reductions after irradiation with IR-laser (810 nm) or LED (457 nm) alone were both marginal and not significant (IR: <0.6 log_10_, 73.627%; LED: <0.8 log_10_, 82.707%). The results show that the drugs are well entrapped in the fibers; ICG mainly serves only to release the CUR, and CUR only works properly when it is released. This reinforces the results in Section 3.4.1 and confirms the superiority of double irradiation and triggered drug release. To visualize the reduction of bacteria, the treated adhered bacteria were analyzed under SEM and the images were presented in Figure 5A–D. The micrographs completely confirmed the previous bacterial assay. In the unirradiated sample, there is a visible accumulation of bacteria on the surface, which are partially conglomerated, as in a biofilm forming (Figure 5A). In contrast, the surface of the double-irradiated sample was almost blank and only a few bacteria were visible (Figure 5B). Both single irradiated samples showed a minimal decrease in adhered bacteria and the conglomerates are also still clearly visible (Figure 5C,D). Although a biofilm is more difficult to treat, as bacteria can separate themselves from the external medium and even antibiotics often cannot penetrate [66], our formulation in combination with double irradiation showed distinct superiority and has an antibacterial effect. In this study, our triggerable formulation was evaluated against gram-positive and gram-negative bacteria only. However, the antibacterial activity of CUR has been successfully tested against many other microorganisms and has been used in multiple clinical studies [19]. For example, the antimicrobial effect of CUR against various Candida species is also often described and proven [67], as is, similarly, its effectiveness against various types of viruses [68]. Currently, CUR is even being studied for its effectiveness in treating hospitalized patients with COVID-19 [69].

The excellent efficacy against various microorganisms and the wide applicability of our formulation could make it suitable for use in many different areas of aPDT (e.g., infected wounds on the skin, gum, or mucous membranes). In addition, an application as an implant could be investigated, as the IR light used to trigger drug release can penetrate deep into the tissue and could enable drug release from outside the body.

### 3.5. Biocompatibility Studies

The cellular biocompatibility of the nanofiber formulations was investigated using L929 cells and is presented in Figure 5F [27,70]. All formulations showed no to low toxicity, with the lowest survival rate of 75.8% observed for the unloaded nanofibers, even if the biocompatibility and harmlessness of PLA were confirmed years ago [71]. However, due to variable cell growth, the standard deviation was relatively high, this was therefore still within the acceptable range. In addition, the fiber structure makes it difficult to always keep all cells for detection, so cell loss could also occur due to the implementation. Other fiber samples tended to show higher cell viability than without fibers. This could be due to the fibrous structure, which mimics tissue and the cells can settle three-dimensionally into the structure and grow well. This effect has often been described, and it promotes wound healing on the skin. In general, no toxicity was observed for the produced formulations, and their use is considered safe.

## 4. Conclusions

We developed a PLA nanofibrous mesh loaded with ICG and CUR for an additive antimicrobial effect that combines the advantages of photothermally triggered drug release by IR-mediated photothermal therapy and physical damage by photodynamic therapy. The mesh was successfully produced by single-needle electrospinning, and its morphology and physicochemical properties were characterized by various analytical methods. All fiber meshes had a smooth surface, were homogeneous in size, and were free of crystals or beads. The loaded fibers had a substantially smaller fiber diameter than the unloaded fibers, which is advantageous. The drug release was photothermally triggerable, and almost no release was measurable after irradiation. This indicates good incorporation of the drugs into the fibers, and may also enable long-term treatment. The temperature of the surrounding medium could be raised to a human tolerable 44.8 °C in a few minutes and be maintained above 38.8 °C for over 30 min. Our treated formulation showed a bacterial reduction of gram-positive and gram-negative bacteria. Using double irradiation of our formulation, we were able to reduce the bacterial viability of *S. saprophyticus* by ~4.4 log_10_ (99.996%). In addition, a generated adhered bacterial film was reduced by >4.3 log_10_ (99.995%), which was visually confirmed with SEM micrographs. Finally, the biocompatibility was confirmed by the viability of L929 cells. In summary, this work illustrates the feasibility of a new antimicrobial system with a controllable release profile that can eradicate pathogens and that could be applied in the local treatment of infected wounds on the skin, gums, or mucous membranes to minimize common systemic side effects of antibiotic treatment.

## Figures and Tables

**Table 1:** Poly(d,l-lactide) (PLA) content (relative to the mass of total spinning solution), indocyanine green (ICG) content (relative to PLA mass), curcumin (CUR) content (relative to PLA mass) of the prepared nanofibers.

Sample Name	PLA Content	ICG Content	CUR Content
PLA.NF	6 wt%	0 wt%	0 wt%
PLA.NF.ICG	6 wt%	3 wt%	0 wt%
PLA.NF.CUR	6 wt%	0 wt%	10 wt%
PLA.NF.ICG.CUR	6 wt%	3 wt%	10 wt%

**Table 2:** ICG and CUR concentration of the prepared nanofibers with their mean fiber diameter.

Sample Name	ICG Concentration ± SD [µg/cm^2^]	CUR Concentration ± SD [µg/cm^2^]
PLA.NF	0	0
PLA.NF.ICG	6.30 ± 0.50	0
PLA.NF.CUR	0	30.46 ± 0.83
PLA.NF.ICG.CUR	6.36 ± 0.16	23.23 ± 0.50

## Data Availability

The data can be shared upon request.

## Supplementary Materials

The following supporting information can be downloaded at: https://www.mdpi.com/article/10.3390/pharmaceutics15020327/s1, Figure S1. Absorption of uric acid at different irradiation times with blue LED (457 nm) of PLA.NF.ICG.CUR, PLA.NF.ICG, PLA.NF.CUR, and PLA.NF. Figure S2. Confocal laser scanning microscope images of fibers loaded with indocyanine green and curcumin (PLA.NF.ICG.CUR) (A-D) and fibers only loaded with curcumin (PLA.NF.CUR). An excitation wavelength of λ_ex_ = 488 nm was used. Scale bars represent 5 µm (A–E) and 20 µm (F).
