# Supplementary material for: Photothermally Controlled Drug Release of Poly(d,l-lactide) Nanofibers Loaded with Indocyanine Green and Curcumin for Efficient Antimicrobial Photodynamic Therapy
Source: Pharmaceutics. 2023 Jan 18;15(2):327. doi: 10.3390/pharmaceutics15020327 (PMC9963466; doi:10.3390/pharmaceutics15020327)
Supplement: Supplementary file 1 [file pharmaceutics-15-00327-s001.zip › pharmaceutics-2141282-supplementary.pdf]

pharmaceutics

MDPI

Supplementary Materials

Photothermally Controlled Drug Release of Poly(D,L-lactide) Nanofibers Loaded with Indocyanine Green and Curcumin for Efficient Antimicrobial Photodynamic Therapy

Bernd Gutberlet †, Eduard Preis †, Valeri Roschenko and Udo Bakowsky *

Department of Pharmaceutics and Biopharmaceutics, University of Marburg, Robert-Koch-Str. 4, 35037 Marburg, Germany

* Correspondence: ubakowsky@aol.com

† These authors contributed equally to this work.

Materials and methods

Singlet oxygen generation

Uric acid (UA) was used for the detection of generated singlet oxygen species since the absorbance would decrease at 290 nm when UA reacted with singlet oxygen species. The nanofiber mesh was precisely cut into round pieces with a diameter of 16 mm. All samples were separately fixed on the bottom of a 6-well cell culture plate (TC plate, Standard, F, Sarstedt AG & Co. KG, Nümbrecht, Germany). Subsequently, 1.5 mL uric acid solution (100 μM in PBS (pH 7.4) with 1% Tween80) was added to the well. After 30 min of incubation under light exclusion and at RT the fibers were irradiated with the IR laser module (810 nm) for 30 min. Afterwards, the well was irradiated with blue LED (λ = 457 nm, 100 mA) in 3 min intervals, respectively. 150 μL samples were transferred to a 96-well plate (Quarz-microplate 96-well Hellma GmbH & Co. KG, Müllheim, Germany) and the absorbance of UA was determined with a microplate spectrophotometer (Multiskan GO, Thermo Scientific, Waltham, MA, USA) at λ = 290 nm in between each interval. Absorbance was plotted against irradiation time.

Confocal laser scanning microscopy (CLSM)

The distribution of curcumin (CUR) was qualitatively analyzed using a confocal laser scanning microscope (CLSM). Briefly, fibers loaded with indocyanine green (ICG) and CUR (PLA.NF.ICG.CUR) and fibers only loaded with CUR (PLA.NF.CUR) were placed on microscope slides without aluminum foil and covered with cover glasses. Images were taken with Zeiss Axio Observer Z1 equipped with an LSM 700 confocal unit (Carl Zeiss Microscopy GmbH, Jena, Germany). Images were recorded using an excitation wavelength of λ_{ex} = 488 nm and appropriate filters.

Results and discussion

Singlet oxygen generation

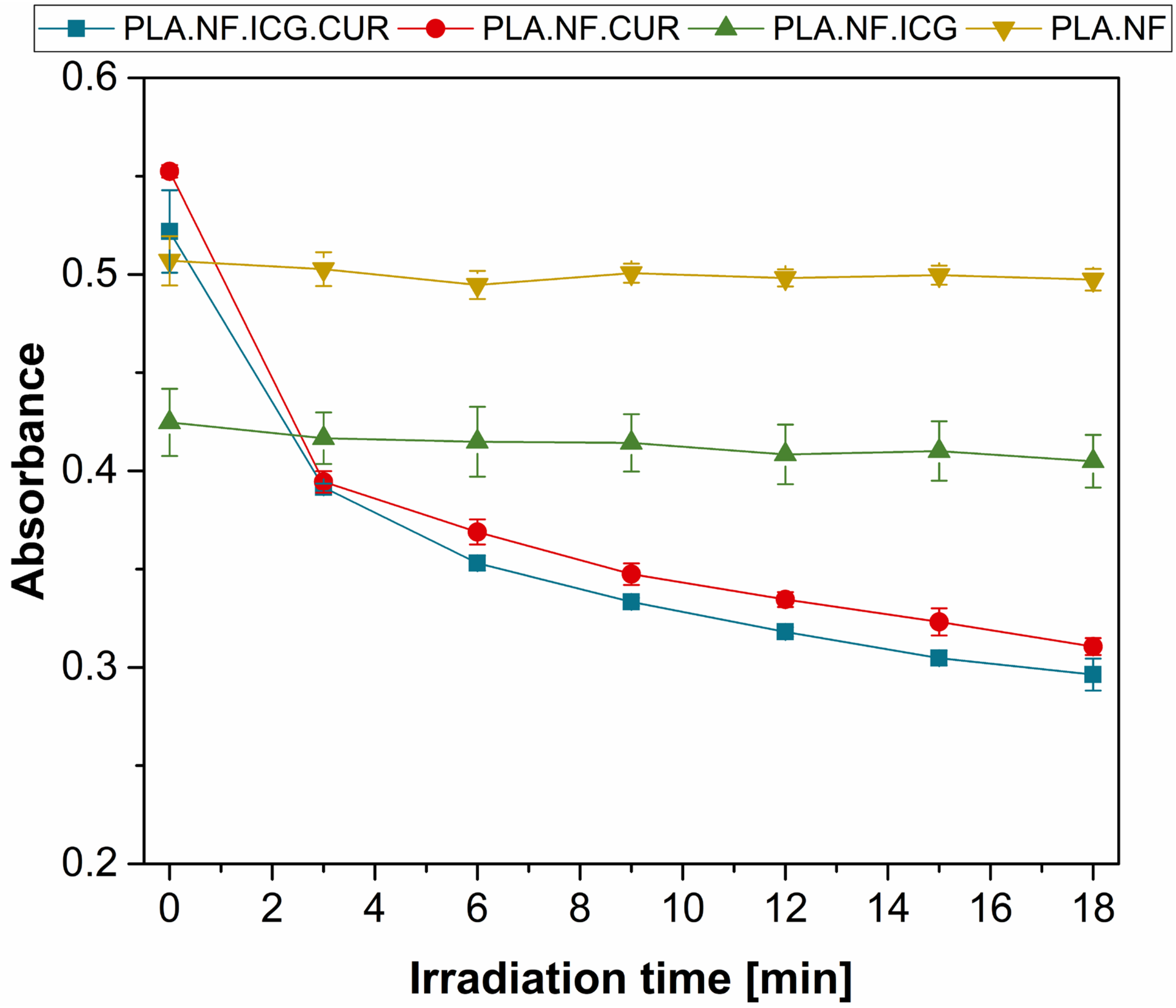

Figure S1. Absorption of uric acid at different irradiation times with blue LED (457 nm) of PLA.NF.ICG.CUR, PLA.NF.ICG, PLA.NF.CUR, and PLA.NF.

Confocal laser scanning microscopy (CLSM)

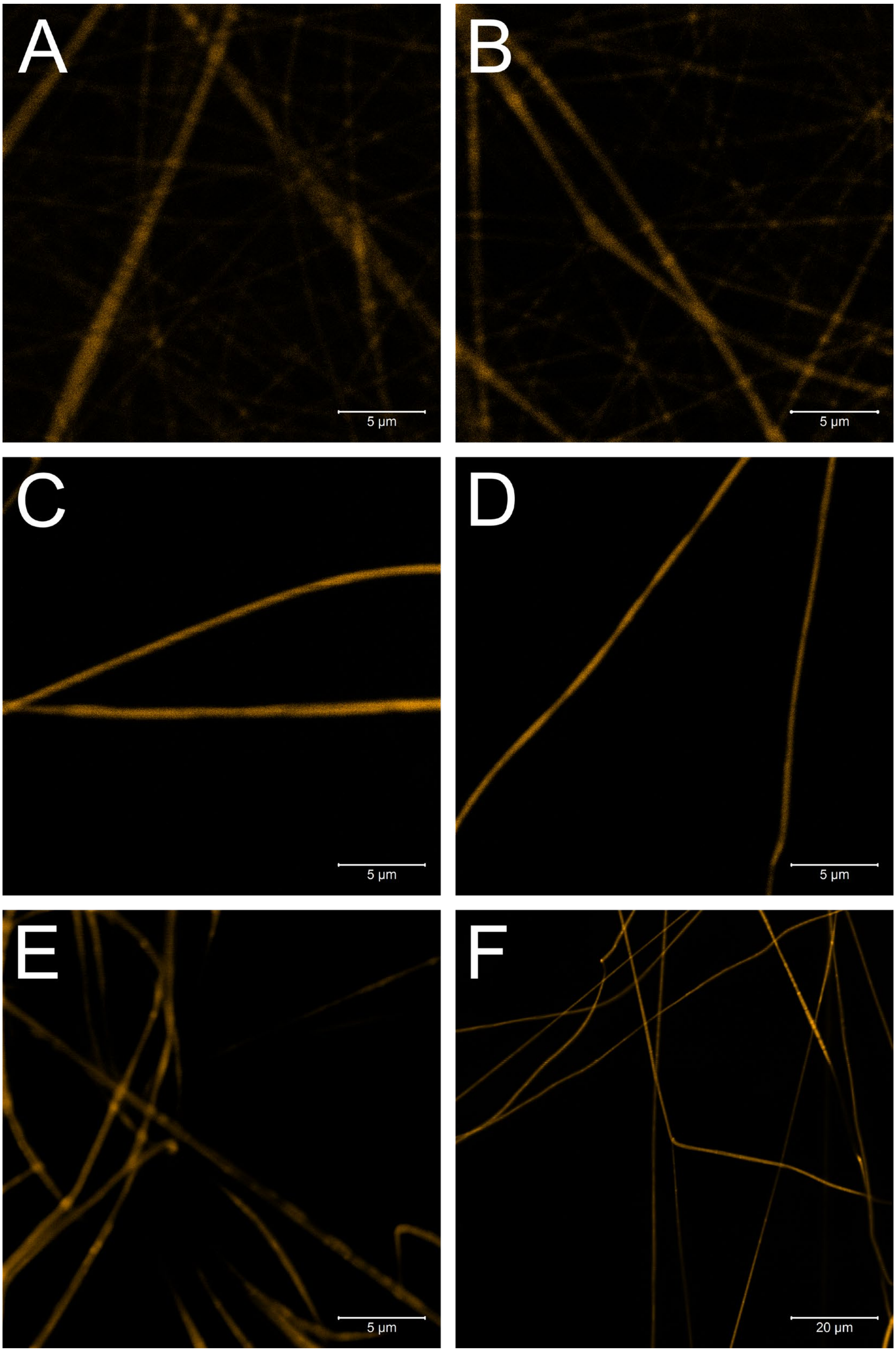

Figure S2. Confocal laser scanning microscope images of fibers loaded with indocyanine green and curcumin (PLA.NF.ICG.CUR) (A-D) and fibers only loaded with curcumin (PLA.NF.CUR). An excitation wavelength of λ_{ex} = 488 nm was used. Scale bars represent 5 µm (A–E) and 20 µm (F).
